# Identification of single motor units in skeletal muscle under low force isometric voluntary contractions using ultrafast ultrasound

**DOI:** 10.1038/s41598-020-79863-1

**Published:** 2020-12-24

**Authors:** Robin Rohlén, Erik Stålberg, Christer Grönlund

**Affiliations:** 1 Department of Radiation Sciences, Biomedical Engineering, Umeå University, Umeå, Sweden; 2 Department of Clinical Neurophysiology, Institute of Neuroscience, Uppsala University, Uppsala, Sweden; 3 Department of Neurosciences, University Hospital, Uppsala, Sweden

**Keywords:** Electromyography - EMG, Biomedical engineering, Ultrasound, Neurophysiology

## Abstract

The central nervous system (CNS) controls skeletal muscles by the recruitment of motor units (MUs). Understanding MU function is critical in the diagnosis of neuromuscular diseases, exercise physiology and sports, and rehabilitation medicine. Recording and analyzing the MUs’ electrical depolarization is the basis for state-of-the-art methods. Ultrafast ultrasound is a method that has the potential to study MUs because of the electrical depolarizations and consequent mechanical twitches. In this study, we evaluate if single MUs and their mechanical twitches can be identified using ultrafast ultrasound imaging of voluntary contractions. We compared decomposed spatio-temporal components of ultrasound image sequences against the gold standard needle electromyography. We found that 31% of the MUs could be successfully located and their firing pattern extracted. This method allows new non-invasive opportunities to study mechanical properties of MUs and the CNS control in neuromuscular physiology.

## Introduction

The central nervous system controls human locomotion by successive recruitment of motor units (MUs) in the skeletal muscles. The MU comprises a motoneuron and a bundle of innervated muscle fibers within a localized territory^1–3^. Today, the function of MUs is measured and analyzed by electromyographical (EMG) methods. It provides the basis for MU analysis^2^ in diagnosing neuromuscular diseases^3^, exercise physiology and sports^4^, and rehabilitation medicine^5^. The EMG records the muscle fibers’ repeated electrical depolarizations following a firing pattern transmitted from the spinal cord via the motor neuron. The so-called excitation–contraction mechanism links the electrical depolarization to the thickening and shortening of the fibers (mechanical twitch). Therefore, recording and analyzing mechanical twitches may provide an alternative way to study MUs.

Non-invasive ultrasound imaging allows mechanical information from a large field of view in soft tissues such as the muscles^6^. In skeletal muscle applications, ultrasound has mainly been used for structural imaging. For example, in diagnostics of neuromuscular disease^7–9^, the quantification of muscle function in exercise physiology and sports^10^, detection of contraction onset^11–13^. High-resolution ultrafast ultrasound imaging^14^, with a high frame rate (> 1000 images per second), has been successfully applied on imaging the MUs’ mechanical twitches during externally controlled *electro-stimulations*^15,16^. Electrical stimulation provides no information about (neural) firing pattern. *Voluntary contractions* are required to access this information. Although more than 10 years have passed since imaging MUs’ mechanical twitch using *electro-stimulation* was published^15^, this has not been shown in *voluntary contractions*. Our group recently proposed a method to extract the mechanical twitches of contracting MUs in ultrasound image sequences under voluntary contractions^17^. The method decomposes the (mechanical) tissue velocity image sequence of the contracting muscle. Using a simplified MU-based biomechanical simulation model^17^, we indicated that the extracted components correspond to mechanical twitches of single MUs. However, the question remains whether the mechanical twitches of a contracting MU can be extracted in real voluntary experimental conditions. The challenge is related to a complex mechanical interaction between the muscle fibers and the skeletal muscle fascia^18,19^ that may influence the identifiability of mechanical twitches.

This study aimed to evaluate if single MUs and their mechanical twitches can be identified in experimental skeletal muscle voluntary contractions. For this purpose, we compared the position and firing patterns of the decomposed components from ultrafast ultrasound acquisitions with the simultaneous needle-EMG recordings (MU action potentials).

## Results

### Synchronized needle-EMG and ultrasound measurements

**Figure 1:**
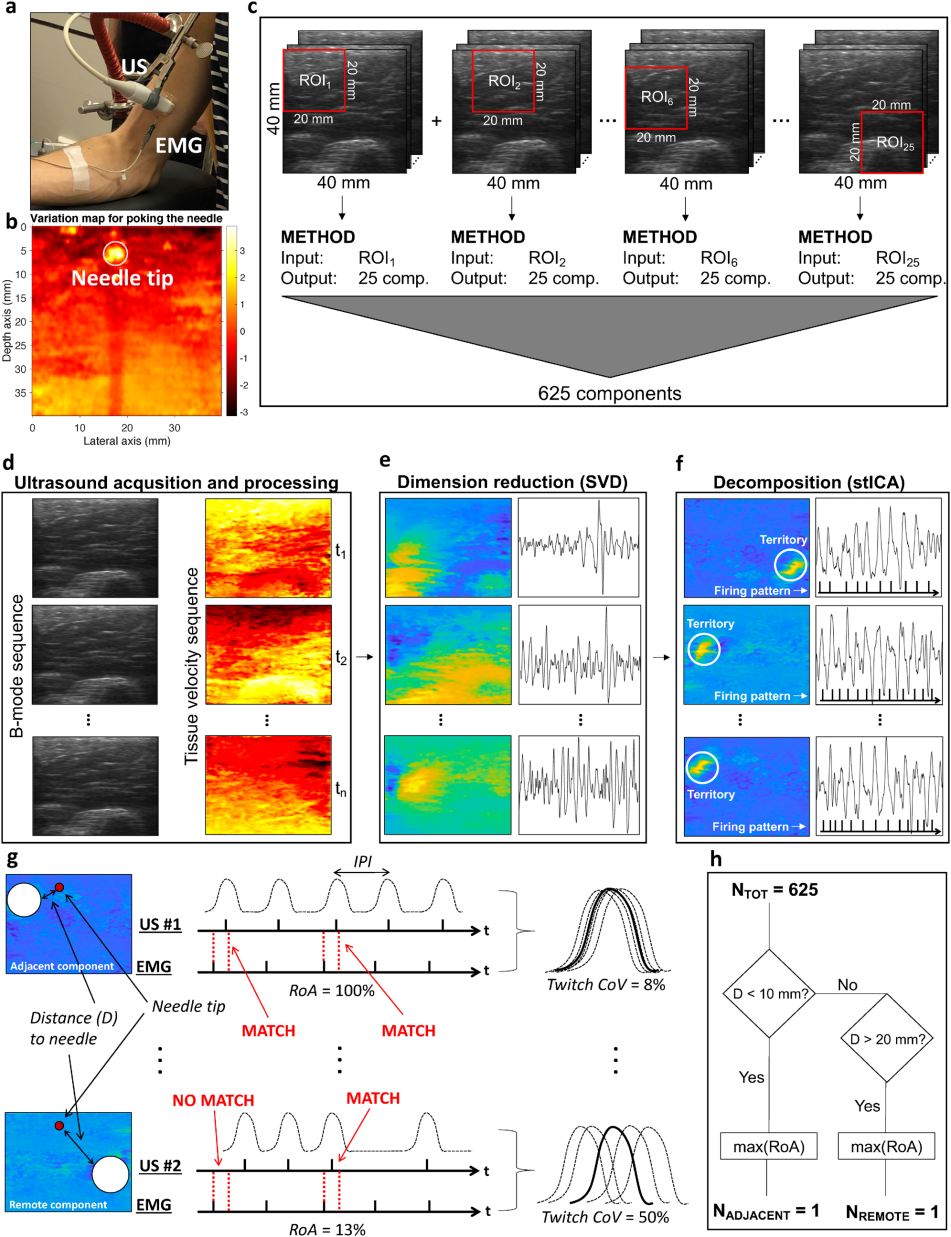
Synchronized ultrasound and needle-EMG measurements were used to compare components’ mechanical twitches with MUAPs. (**a**) Measuring with ultrasound (US) and needle-EMG simultaneously on the biceps brachii. (**b**) Identification of the needle tip by poking the needle and calculating a pixel-wise variation map. (**c**) For each measurement, we applied our decomposition method to 20 × 20 mm windows (on a 40 × 40 mm field of view) of ultrasound data to extract a total of 625 components (referred to as comp. in the figure). The bone is seen at the bottom of the images as a semi-circle. (**d–f**) An illustration of the method to identify the MUs’ mechanical twitches using singular value decomposition (SVD) and spatio-temporal independent component analysis (stICA). (**g**) An illustration of the different features (*IPI *inter-pulse-interval, *RoA *Rate of Agreement, *Twitch* *CoV *Twitch Coefficient of Variation) to study similarities of the components and the MUs. (**h**) These 625 (N_TOT_) components were stripped down to two components (based on distance D mm to needle-tip and its maximal RoA) being the adjacent (N_ADJACENT_ = 1) and remote component (N_REMOTE_ = 1).

We collected 68 EMG- and ultrasound synchronized measurements (from a total of nine subjects in biceps brachii muscle). Four of these measurements were excluded due to EMG-, ultrasound-, or synchronization errors and left us with a total of 64 EMG- and ultrasound synchronized measurements (Fig. 1a). The (concentric) needle electrode tip locations were identified in the ultrasound image sequences by poking the needle (Fig. 1b). All needle tip locations in the ultrasound field of view are shown in Supplementary Fig. S1. The needle-EMG measured MU action potential (MUAP) features (duration, amplitude, and firing rate) in the 64 EMG recordings (Table 1) were in line with values of biceps brachii in normal subjects^20^.

**Table 1:** Bioelectrical features recorded by needle-EMG are in line with the values of biceps brachii in normal healthy subjects.

Feature	Value
MUAP duration (ms)	15.1 ± 4.0 (6.5; 23.0)
MUAP amplitude (mV)	0.29 ± 0.28 (0.05; 1.64)
MU firing rate (Hz)	11.0 ± 2.2 (6.9; 17.1)

The features correspond to the MUAPs duration, amplitude, and the MU’s firing rate in the biceps brachii for the 64 MUs.

### Comparison of decomposed spatio-temporal components from ultrasound with MUs from EMG

The ultrasound data were processed by sliding an 20 × 20 mm region-of-interest (ROI) over the full field of view (40 × 40 mm) (Fig. 1c). This process resulted in 625 *decomposed spatio-temporal components* for each EMG- and ultrasound synchronized measurement (Fig. 1c) by calculating tissue velocity sequences and applying a decomposition method^17^ (Fig. 1d–f). We selected one adjacent- and one remote component (referring to < 10 mm and > 20 mm from needle tip) out of the 625 components per measurement (Fig. 1g,h). The selection of components was based on (1) the rate of agreement between components’ and the MU firing pattern (RoA) and (2) the components’ twitch variability with respect to MU firing pattern (twitch CoV). Thus, we had 64 paired components (one adjacent and one remote component), for all 64 EMG- and ultrasound synchronized measurements. The purpose of selecting one adjacent and one remote component per measurement was to let the remote components act as a control group to the adjacent components since the MU is surrounding the needle tip. Thus, the adjacent components are selected candidates of identified MUs.

**Figure 2:**
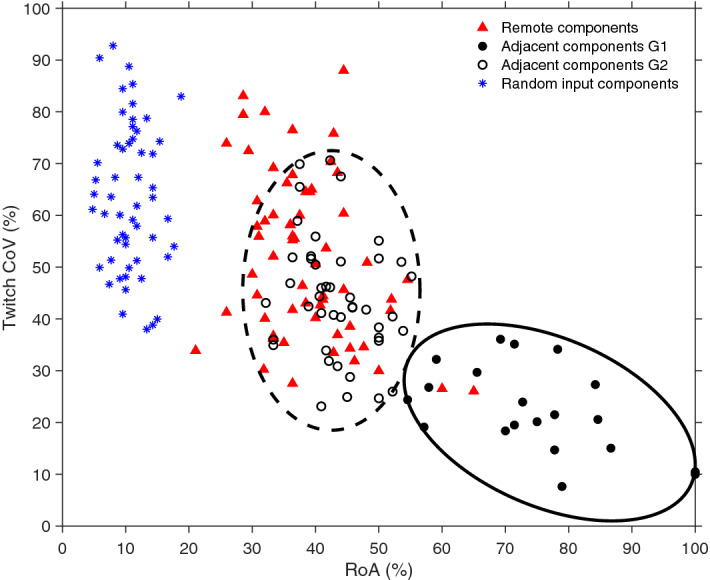
Successful identification of MUs were based on RoA (the rate of agreement between component and MU firing patterns) and twitch CoV (twitch variability with respect to MU firings). Remote components (triangles), adjacent components (circles), and random input components (asterix) are visualized. The adjacent components (circles) are divided into two groups, i.e., adjacent components G1 (filled circles) and adjacent components G2 (unfilled circles) by using K-means method. The two groups are illustrated using ellipses. Asterix (blue) corresponds to components from random noise input to the decomposition method.

We observed a large range of RoA and twitch CoV (Fig. 1g) for the adjacent and the remote components (see Supplementary Table S1). We divided the 64 adjacent components into two groups using K-means (K = 2) based on the RoA and twitch CoV variables (Fig. 2). This resulted in one group that overlaps the remote components (referred to as adjacent components G2) and one group with no overlap (referred to as adjacent components G1). Adjacent components G1 included 20 of the 64 adjacent components (31%) and had a high RoA (74.6 ± 12.7%) and low twitch CoV (22.3 ± 8.5%). Adjacent components G2 included 44 of the 64 adjacent components (69%) and had a lower RoA (43.5 ± 5.8%) and a higher twitch CoV (44.2 ± 11.7%). The remote components also had a low RoA (38.5 ± 8.1%) and a high twitch CoV (53.7 ± 22.4%). These values are shown in Supplementary Fig. S2 and in Supplementary Table S1.

**Figure 3:**
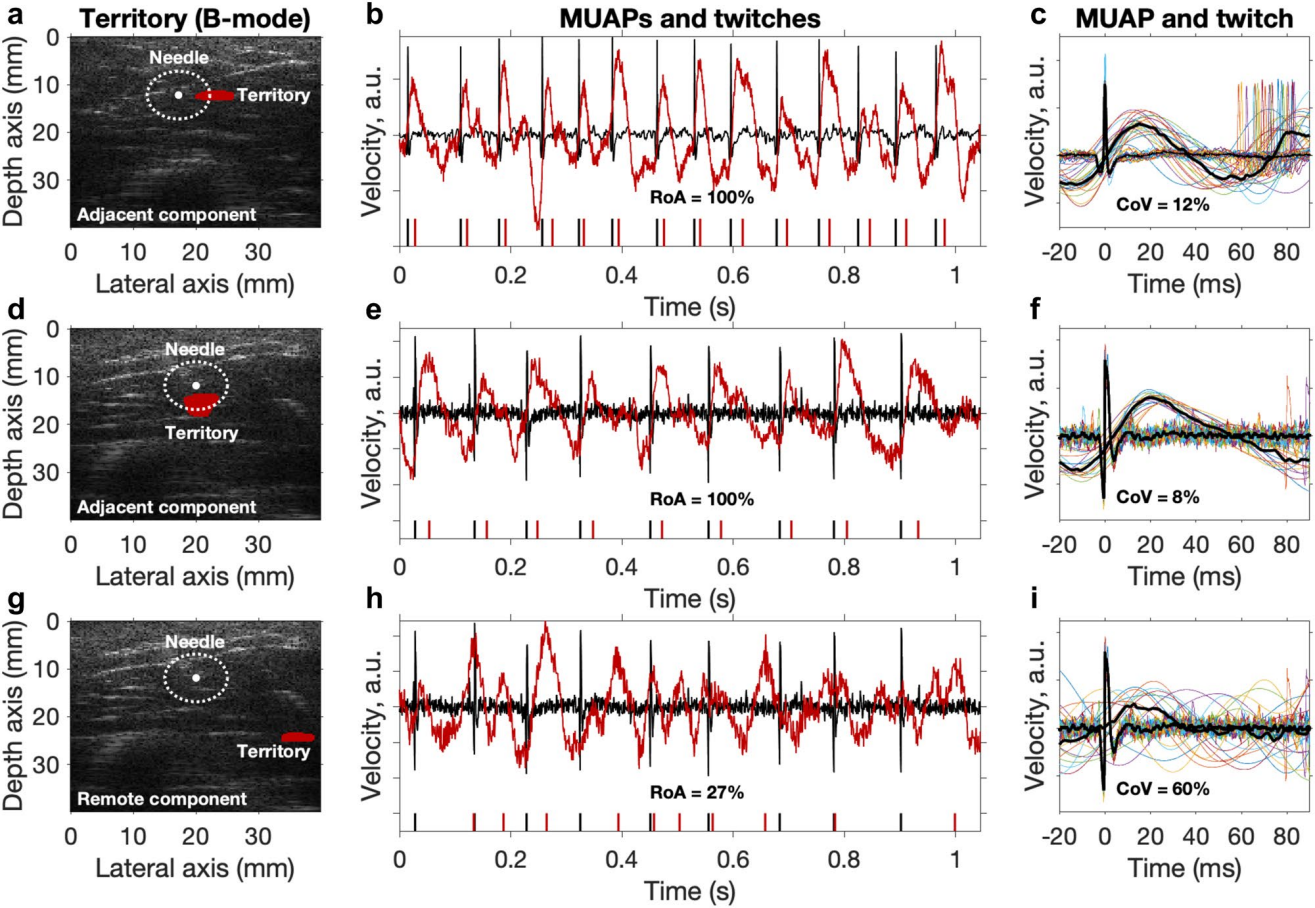
Three examples of decomposed components (territory and twitches) from ultrafast ultrasound together with MUAPs from EMG. The first two rows (**a**–**c**,** d**–**f**) are adjacent components and the last row (**g–i**) is the corresponding remote component to (**d–f**). (**a**,**d**,**g**) Components’ MU territory (red region) of biceps brachii cross-section and the concentric needle uptake area (white dotted circle). (**b**,**e**,**h**) The MUAPs (black), components’ twitches (red), and their corresponding firing patterns below (black and red vertical lines). (**c**,**f**,**i**) The spike-triggered averaged mechanical twitches given the MU firing pattern (from EMG).

Figure 3 shows three examples of components and the corresponding MUAPs from needle-EMG. Two examples are adjacent components (close to the needle tip) having a high RoA and low twitch CoV, whereas the third example is a remote component far away (> 20 mm) from the needle tip having a low RoA and high twitch CoV. Note that the remote component (Fig. 3g–i) is from the same dataset as the second adjacent component (Fig. 3d–f). For the first two (adjacent) components, their mechanical twitch was delayed relative to the MUAP (Fig. 3c,f) as expected due to the electromechanical delay.

In summary, the components’ firing patterns and positions were highly similar to the MUs’ recorded by EMG in 31% of the EMG- and ultrasound synchronized measurements. From now on, we refer to the adjacent components G1 as successfully identified mechanical twitches of single MUs.

### Assessment of subject dependencies

**Figure 4:**
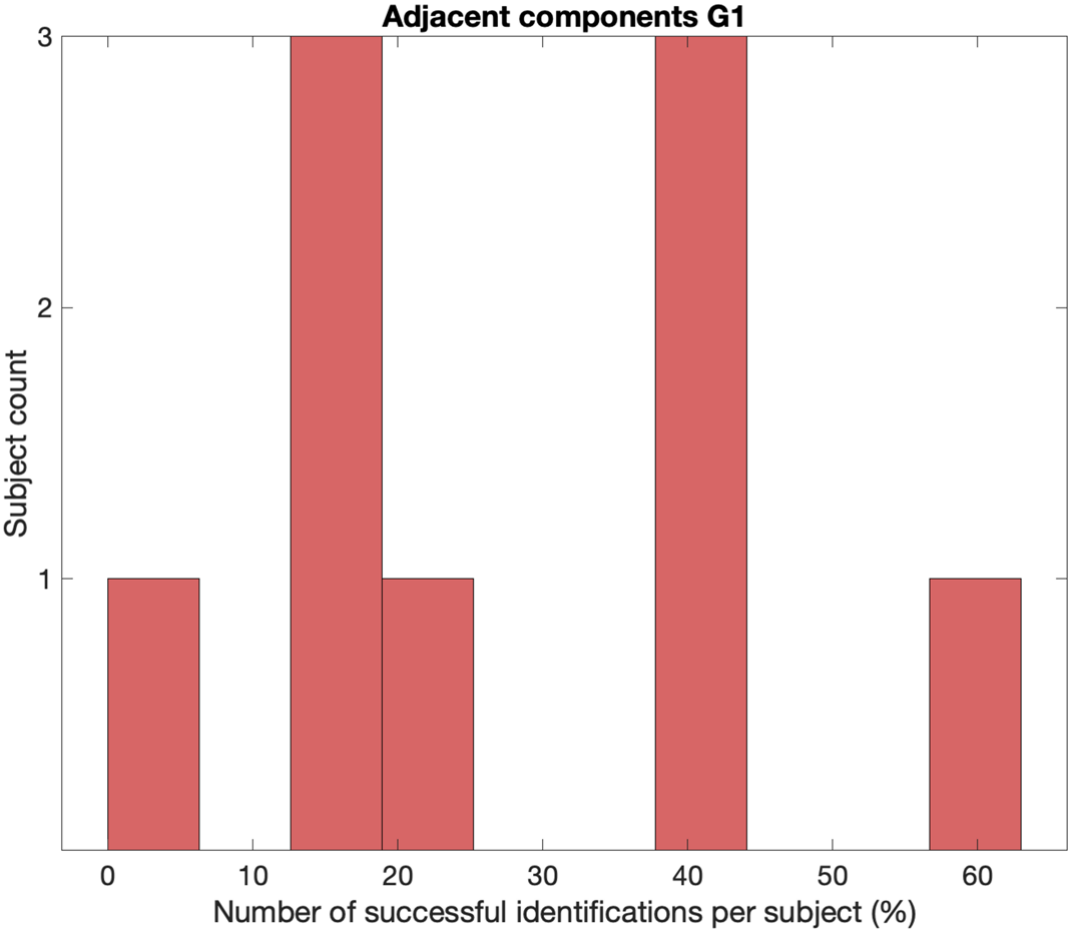
Disproportionate representation in subjects’ successful identification of MUs suggests subject-specific dependency. Successful identification of MUs varied between subjects (0–63% of the measurements per subject). If there would not exist a subject dependency in identifying MUs, the successful identification of MUs should have a small variation around a particular percentage.

We found that successful identification of MUs varied between subjects (0–63% of the measurements per subject). If there is no subject dependency in identifying MUs, the successful identification of MUs should have a small variation around a particular percentage point. However, in 4 of 9 subjects (44%), we obtained > 40% successful measurements per subject, whereas the other five subjects (56%) had < 20% successful measurements per subject (Fig. 4). This result indicates a disproportionate representation of subjects and a subject-specific dependency (see Supplementary Table S2).

In summary, the successful identification of the MUs’ mechanical twitches may be subject-dependent.

### Case with an observation of distributed MU territory

**Figure 5:**
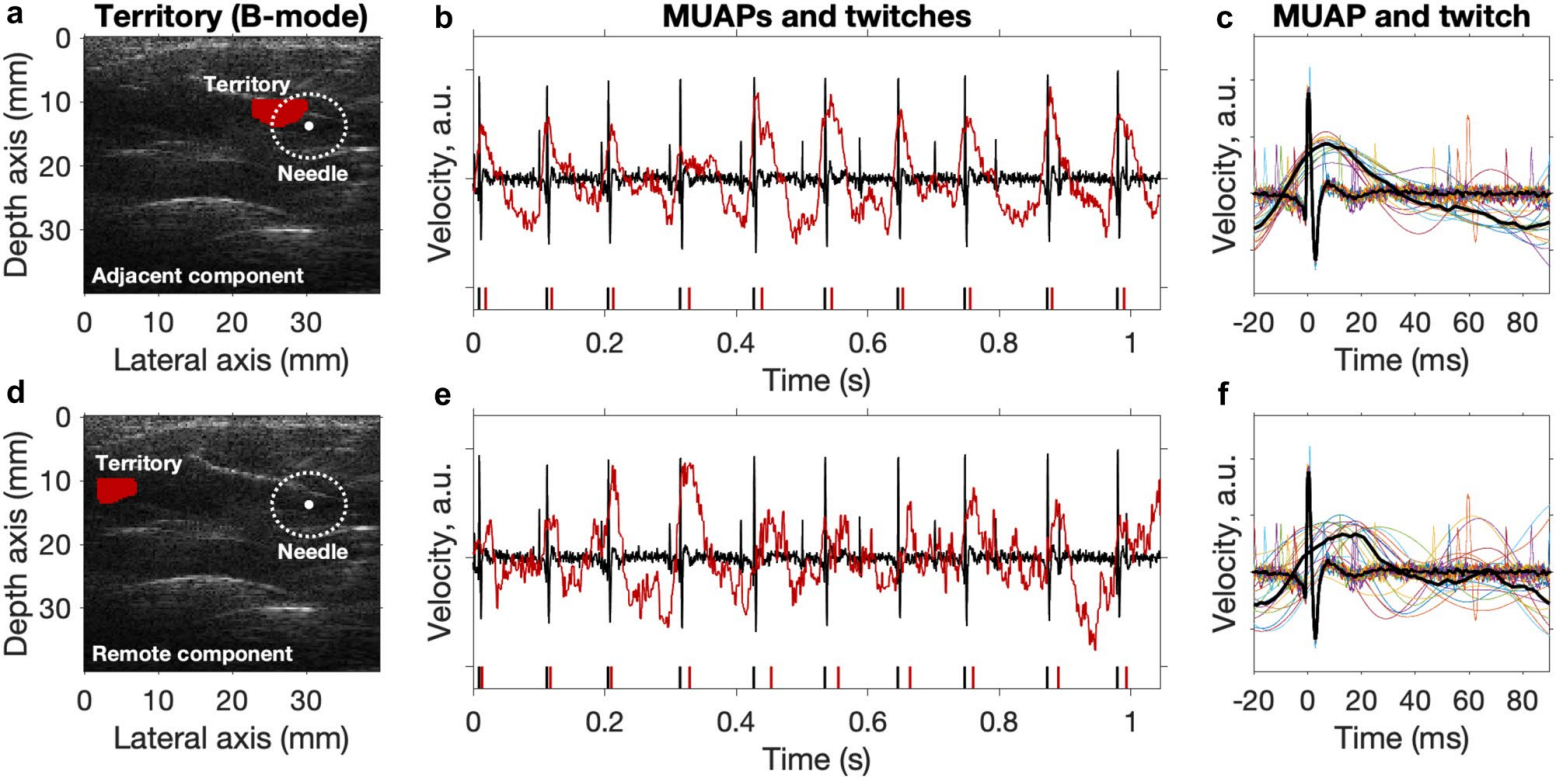
Observation of similar RoA and twitch CoV indicate distributed MU territory (for the same MU). (**a**,**d**) Components’ MU territory (red region) of biceps brachii cross-section for one subject with an adjacent and remote component. The white dotted circle corresponds to the concentric needle uptake area. (**b**,**e**) The MUAPs (black), components’ twitches (red), and their corresponding firing patterns below (black and red vertical lines). (**c**,**f**) The spike-triggered averaged mechanical twitches given the MU firing pattern (from EMG).

We found two remote components (> 20 mm from the needle tip) with high RoA and low twitch CoV within the adjacent components G1 (Fig. 2). We show one example of these remote components’ territory, mechanical twitches, and the corresponding MUAPs together with their adjacent components in Fig. 5 (both examples are shown in Supplementary Fig. S2). Interestingly, these remote components’ RoA and twitch CoV was similar to the adjacent components (Fig. 5b,c,e,f). These observations may indicate that these two territories are controlled by the same innervating motor neuron (i.e., the same MU), although they are 20 mm apart (Fig. 5a,d). This finding is in contrast to previous studies showing that MU territories are considered to be localized^21,22^. Both measurements come from the same subject. Identifying the same territories in two measurements from the same person indicates the repeatability of the method (see Supplementary Fig. S2).

In summary, in two measurements, we found two components with territories far apart (> 20 mm) with similar RoA and twitch CoV.

### Influence of random input

The method uses sliding ROIs and multiple overlapping windows for processing, which could potentially be due to the multiple-testing concept produce false positives agreeing with the MUs from EMG. To evaluate the risk of this effect, we simulated random white noise and used it as input to the method (Fig. 1c–f). We found that these artifact noise components produced a group of its own compared to both adjacent and remote components (Fig. 2) since they have a low RoA (11.6 ± 2.7%) and high twitch CoV (80.8 ± 87.5). See Supplementary Table S1.

In summary, the method does not produce artificial false positive components when random noise is input.

## Discussion

In this work, we aimed to identify single MUs and their mechanical twitches in ultrasound image sequences of voluntary skeletal muscle contractions. We compared decomposed spatio-temporal components from ultrasound image sequences against MUs from the gold standard needle-EMG. The main finding is that the components’ positions and firing patterns were highly similar to 31% of the MUs. About 44% of the subjects accounted for the majority of the 31% successful identifications. The results imply (1) the firing pattern and position of single MUs can be identified in skeletal muscles under low force voluntary contractions, (2) the successful identification of the MUs’ mechanical twitches may be subject-dependent, and (3) the decomposition of ultrasound image sequences provide a means to study MU position better than other methods.

The MUs’ mechanical twitches could be detected in 31% of the EMG- and ultrasound synchronized measurements but not in 69% of them. There are five points relevant to these findings that need to be discussed.

1. Previous results support the finding that the mechanical twitches of single MUs can be identified by mechanomyography (MMG). There are indications that the mechanical twitches of contracting MUs superimpose linearly under low force levels with a limited number of MUs activated and at low firing rates^23–25^ but not at high force contractions involving many MUs. At higher firing rates, a non-linear summation of the twitches likely occurs.
2. Even though our measurements were at low force levels and low firing rates, identification failed in 69% of the measurements. These findings can be related to the inter-subject variability of successful measurements, which could be explained by an inter-subject difference in the mechanical properties of the perimysium (fascicle level) of the skeletal muscle fascia^18,19^. In particular, glucosamine (hyaluronic acid) promotes muscle deformation and gliding^26^ at the interfaces between the muscle fascial structures. This material has thixotropic properties^27^, meaning, thick or viscous under static conditions and thinner or fluidic under dynamic conditions. We did not apply standardized warmup protocol, and thus the thixotropic state might have varied between subjects. Both these mechanisms could contribute to an observed inter-subject variation and variation in detecting the MUs’ mechanical twitches.
3. The choice of the decomposition method could also influence the ability to identify the MUs’ mechanical twitches successfully. The applied decomposition method relies on an assumption of linearly super-positioned components. It achieves separation by maximizing spatial and temporal distribution features of the components (without any dependency of or order or position of time samples and spatial pixels). However, using a more complex decomposition model, considering more a priori assumptions (both temporal and spatial), the rate of successful identification of MUs’ mechanical twitches may be improved.
4. The concentric needle-EMG may also influence the successful identification of MUs’ mechanical twitches in two ways: (A) The tip of the needle must be precisely localized to match correct component as adjacent component. Since we used a ± 5 mm region, we believe that this is not a major problem. (B) The needle is stiff and, when inserted, may act as a mechanical bridge between the investigated muscle segment, its adjacent muscle tissue, and the penetrated skin, and cause an attenuation of the mechanical twitches of a MU. However, we could not identify any difference in the magnitude of the TVI variation (see Supplementary Table S1), which indicates that the attenuation should be weak.
5. No motion correction was applied to the data. Since the decomposition relies on a pixel-to-tissue mapping, e.g., large probe movements could corrupt the data. However, due to the experimental protocol’s stable isometric contraction, probe holder, and relatively short acquisition time, we do not believe that this had any large influence on the results. Motion correction should be useful in potential future applications of the method, e.g., dynamic contractions.

Several of the method’s parameters are the same as in Rohlén et al.^17^, where they were empirically optimized for simulation data, and their impact on performance was also discussed. The impact of these parameters on the decomposition may differ in the present work since we deal with experimental data. In particular, the firing pattern estimation from the twitches includes a threshold to identify the firings. Non-random noise or other physiological influence (e.g., motions) could have influenced the identified number of firings, which in turn could have influenced the RoA computation. In the future development of this technique, the firing pattern estimation should be optimized. Moreover, the original method also used the whole field of view (40 × 40 mm) as input. Here, we used a sub-region (20 × 20 mm, 64 × 64 pixels). The motivation to use a smaller field of view was to increase the validity of the assumption of a linear combination of sources in the decomposition method. At the same time, it still has an ROI large enough to include the MU territory (2.5–10 mm diameter biceps).

In the original version of the method^17^, a component selection step was integrated and trained on simulated data to automatically output estimated mechanical twitches. Here we omitted this step to minimize the risk of selection bias and account for differences between simulated and experimental data. Thus, not telling if the MUs’ mechanical twitches is available in components of the ultrasound image sequences. In future work, a post-processing component selection step for experimental data could be trained. For example, based on the Supplementary Table S1, we assume that the inter-pulse-intervals coefficient of variation (IPI CoV) and a twitch CoV (the components’ twitch variability with respect to the firing pattern) may be suitable variables in a component selection step.

Today, there exist several methods to record and analyze the MU function. The standard method is the EMG, including invasive needle-EMG, non-invasive high-density surface-EMG. Needle-EMG (concentric) typically samples electrical potentials at the tip of a needle 2.5 mm radially in a semi-circle^28,29^. Scanning-EMG is an advanced EMG method^30^ that gives information about fiber distribution with a cross section of the territory, but only in one direction. High-density surface-EMG uses electrode arrays or matrices on the skin’s surface to record the muscle’s electrical activity and decompose signals into single MUs^31,32^ within measurement depths down to 1 cm^33^. Thus, EMG methods have a limited field of view. Recent large field of view imaging-based techniques, such as ultrafast ultrasound^15,16^ and magnetic resonance imaging^34–36^ (MRI), have shown promise to overcome these spatial limitations and access the whole muscle during transcutaneous electro-stimulation. However, electro-stimulation does not provide information about the (neural) firing patterns of MUs and, thus, has limited ability to study the MU function thoroughly. In the present work, we show that the position, neural firing pattern, and mechanical twitches of single MUs can be identified by decomposing ultrafast ultrasound imaging sequences.

This study’s results suggest several potential applications. For example, combining our method’s mechanical information (twitches) of contracting MUs with the corresponding electrical excitation information of EMG methods opens possibilities to study the excitation–contraction coupling. Given that a post-processing component selection step would be added^17,37^ (see above), simultaneously activated MUs and their *firing patterns, positions, and possibly territory sizes* could be identified (without the EMG truth). This large field of view information on simultaneously active MUs allows increased accessibility compared to current methods to study neuromuscular physiology. For example, in the central nervous system’s strategies on MU recruitment^38,39^ and the control of prostheses^40^.

In conclusion, current state-of-the-art methods to study MU function includes needle- and surface-EMG, which are well-established techniques that have been used for over 60 years to help our understanding of muscle physiology and diseases. Here we show, for the first time, that single MUs and their mechanical twitches can be identified in experimental voluntary contractions. The decomposition of ultrasound image sequences provides a non-invasive means to study the neural control of MUs and their mechanical properties with a superior large field of view compared to any currently available methods.

## Methods

### Definitions

A *mechanical twitch* is here the thickening of MU’ fibers after one depolarization, i.e., the EMG analog to the MU action potential (MUAP). *Twitches* is the fusion of repeated twitches caused by the repeated depolarizations of a MU firing pattern, i.e., the EMG analog to the MUAP train (series of MUAPs). A *component* is the spatio-temporal output of an ultrasound decomposition, where the spatial counterpart corresponds to the territory and the temporal counterpart corresponds to the twitches.

### Experimental procedure

Nine healthy subjects (27–45 years old, four men and five women) were seated in a chair and performed low force isometric elbow flexion. The right elbow was positioned on elbow support of the chair’s arm support. A physician inserted a standard clinical concentric needle into the biceps brachii muscle. Guided by the sound of active MUAPs and instructions from the physician, the subject was instructed to generate a steady weak force by supinating the hand while the physician gave feedback based on the recorded MUAP. When the EMG signal showed high signal-to-noise ratio MUAP from one MU, the subject was asked to maintain this activation. The first step was to acquire ultrasound data for two seconds while lightly poking the needle to facilitate localization of the tip of the needle in a post-processing stage^41,42^. The second step was to acquire two seconds of ultrasound data simultaneously with the EMG data (Fig. 1a). A limited window of two seconds was selected to achieve a stable MU recruitment^17^ and to minimize the risk of motion from non-MU activity. The above procedure was repeated for 3–9 MUs for each subject. The subjects gave written informed consent, and the project conformed to the Declaration of Helsinki and was approved by the Swedish Ethical Review Authority (dnr 2019-01843).

### Needle tip identification

To identify the needle tip in the image plane, we calculated tissue velocity images from the poking-recordings. We estimated the variance for each pixel to create a so-called tissue velocity variation map (Fig. 1b). The needle location was then found by identifying the coordinate where we had a small confined circular region.

### Electromyography acquisition

The EMG recordings were performed on a Cadwell Sierra Wave EMG machine (Cadwell Laboratories Inc., Kennewick, WA, USA) sampling at 64 kHz with a 38 × 0.45 mm concentric needle electrode (AMBU Neuroline, DEN).

### Ultrasound acquisition and tissue velocity estimation

A research ultrasound system (SonixTouch, Ultrasonix Medical Corporation, Richmond, CA) was used together with a 9 MHz L9-4 linear transducer and a 128 channel DAQ module, to acquire two seconds of data from a plane wave transmit and parallel receive sequence at an image rate of 2000 images per second^14^. The radiofrequency (RF) data was sampled at 40 MHz and reconstructed using sum-and-delay beamforming method^15^ at a field of view of 40 × 40 mm. Tissue velocity image (TVI) sequences were estimated from the reconstructed RF images using the two-dimensional autocorrelation approach^43^ with 1 mm maximal displacement in the depth direction for all channels between subsequent images and a sliding window of 10 ms. Only the axial component was retained^15^. The reconstructed RF-line signals were band-pass filtered (2–15 MHz with order 6), and a high-pass filter (5 Hz) was applied along the frame time of the velocity data before a spatial 2-D median filtering (1 × 1 mm kernel)^17^. The data was downsampled in the axial direction such that a final spatial resolution or 0.3 × 0.3 mm was achieved.

### Synchronization of modalities

The ultrasound and EMG systems were synchronized using a customized synchronization procedure. An optically isolated pushbutton switch was connected to a + 5 mV DC in parallel on the EMG recording channel and a high-speed switch connected in series with the trigger signal from the main SonixTouch unit output to the scanline trigger input channel of the DAQ module. The master clock of the ultrasound was 50 MHz, and the sample rate of the EMG at 64 kHz.

### Firing pattern estimation MUAP

Detection and classification of MUAPs from the raw EMG data were performed by the method proposed by Stålberg et al.^44^. Before analysis, a low-frequency synchronization signal effect was removed from the EMG signal by a polynomial function.

### Identification of mechanical twitches

The identification of mechanical twitches of single MUs is based on Rohlén et al.^17^. In this study, we modify the original method in two ways. (1) While the original method included a component selection post-processing step (trained initially on simulated data). Here, it was omitted to minimize the risk of selection bias and account for differences between simulated and experimental data. (2) A spatial sub-region of 20 × 20 mm (64 × 64 pixels) was selected to maximize the validity of the assumption of a linear combination of sources as compared to using the full field of view. We slide the window with jumps of five mm in both directions resulting in 25 sub-regions (Fig. 1c).

The processing may be summarized as for each ROI; we reduce the dimension of the 64 × 64 × 4000 data (two seconds at 2 kHz equals 4000 frames) by singular value decomposition (SVD) to simplify for the decomposition algorithm by extracting 25 components (Fig. 1c,e). Then we apply spatio-temporal independent component analysis (ICA) to decompose 25 components, and each component’s territory and firing pattern were estimated. In total, this resulted in 25 × 25 = 625 components from each recording. See Fig. 1d–f for an overview of the method and Rohlén et al.^17^ for specific details.

### Dividing the decomposed components into adjacent and remote components

We divided the decomposed components into adjacent and remote components where adjacent and remote refer to the distance (< 10 mm and > 20 mm) to the needle (Fig. 1h). The purpose of this split was to let the remote components act as a control group to the adjacent components.

### Comparing the decomposed components with MU characteristics

To test whether the components’ mechanical twitches is caused by MU activity, we compared the mechanical twitches with MUAPs from simultaneously collected needle-EMG recordings. In general, single MUs fire independent of each other in the non-fatigued muscle. Therefore, the MUAP can be used to extract synchronized mechanical activity from the same MU uniquely. We hypothesize that the EMG method only provides the activity of one MU at a specific location (1 mm^3^ volume). In contrast, the ultrasound provides a large field of view and all active MUs in that field (4 × 4 cm). Thus, for each synchronized EMG-ultrasound measurement, there could be numerous active MUs, even at weak contraction levels, located outside the limited field of view of the EMG. We chose to solve this problem by comparing all 625 components’ and MU firing pattern and compare components located close to the needle (adjacent) with components located far away from the needle (remote).

For each component’s mechanical twitches, we first compared the position of the components’ territory with the tip of the needle position, *D*, as the (minimum) Euclidean distance. Next, we compared the components’ and MU firing patterns (from EMG) by the RoA descriptor, quantifying the rate of agreement. The RoA was calculated as $RoA=100\times {c}_{j}/{(c}_{j}+{A}_{j}+{B}_{j})$, where ${c}_{j}$ is the number of firings of the *j*th firing pattern that was identified jointly by the component and MU (matched firings), ${A}_{j}$ the number of false identified firings, and ${B}_{j}$ the number of unmatched firings. The tolerance in electro-mechanical delay was set to 30 ms^45^. A twitch coefficient of variation (CoV) was calculated as the mean absolute deviation divided by the median based on spike-triggered averaging the component’s mechanical twitches using the MU firing pattern (from EMG).

Additional features of the mechanical twitches were also computed. The firing rate was calculated as the number of firings in the firing pattern divided by the total time (in seconds), inter-pulse-interval (IPI) was calculated as the mean time between firings (in milliseconds), and TVI variation as the standard deviation of the absolute value of all pixel values. See Fig. 1g for the illustration of the different features.

### Influence of random input

The method uses sliding ROIs and multiple overlapping windows for processing. To evaluate the risk of producing false positives agreeing with the reference method (multiple-testing concept), we simulated standardized Gaussian distributed white noise (zero mean and standard deviation equal to one) and used it as input to the method (Fig. 1c–f). The random white noise was of the same size as the collected data (128 × 128 pixels and 4000 frames equaling 40 × 40 mm and 2 s at 2 kHz).

### Statistical analysis

Due to the exploratory nature of this study, the sample size was not chosen to detect a pre-specified effect size.

### Data processing

All data processing was carried out using MATLAB (2019b, Mathworks, Nattick, MA, USA).

## Supplementary Information

Supplementary Information.

## Data Availability

The data that support the findings of this study are available on request from the corresponding author R.R. The raw data are not publicly available because of the large file sizes.
