# Supplementary material for: Identification of single motor units in skeletal muscle under low force isometric voluntary contractions using ultrafast ultrasound
Source: Sci Rep. 2020 Dec 24;10:22382. doi: 10.1038/s41598-020-79863-1 (PMC7759573; doi:10.1038/s41598-020-79863-1)
Supplement: Supplementary file 1 — Supplementary Information. [file 41598_2020_79863_MOESM1_ESM.pdf]

Supplementary Information

Identification of single motor units in skeletal muscle under low force isometric voluntary contractions using ultrafast ultrasound

Robin Rohlén[1], Erik Stålberg[2], Christer Grönlund[1]

1) Dept. of Radiation Sciences, Biomedical Engineering, Umeå University, Umeå, Sweden
2) Dept. of Clinical Neurophysiology, Dept. of Neurosciences, University Hospital, Uppsala, Sweden

Supplementary Information

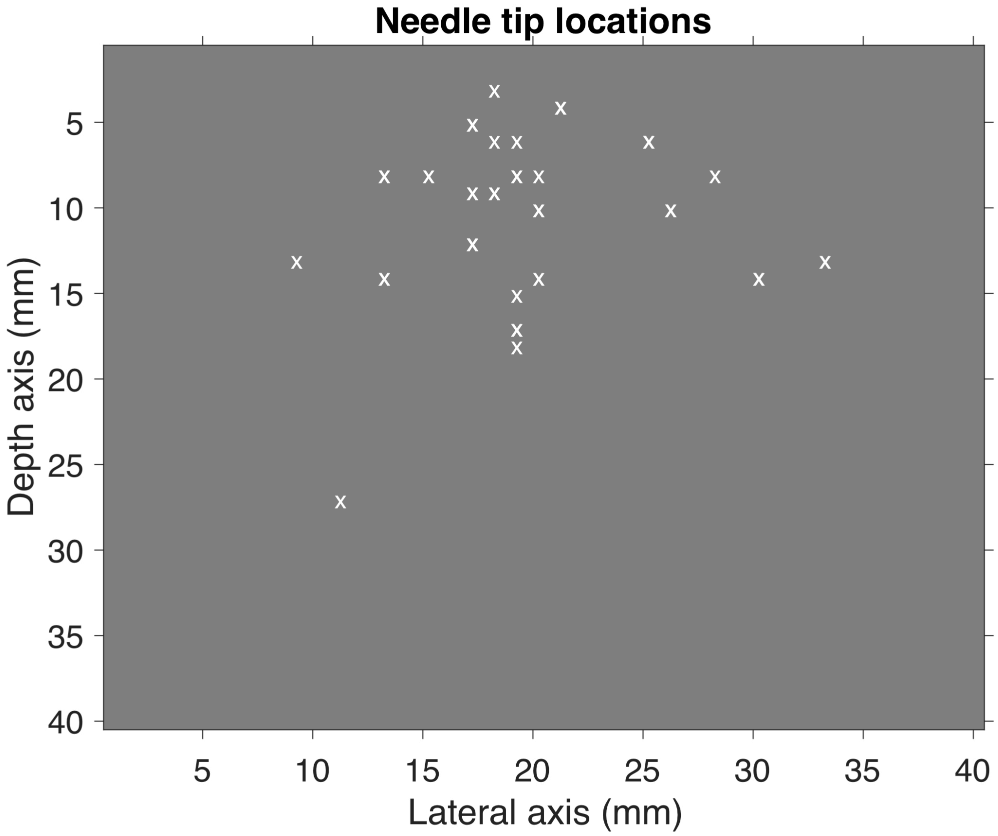

Figure S1 Needle tip locations for all 64 synchronized EMG- and ultrasound measurements where some locations are the same for multiple measurements.

Table S1 A features comparison of components (from ultrasound) and motor units (from EMG) grouped by adjacent and remote location to the concentric needle electrode. Last column is descriptive statistics for components from simulated white noise that has gone through the same methodological procedure as the acquired ultrasound data. These components are compared to the corresponding number for MU (#1 to #64).

	Adjacent components D < 10 mm	Adjacent components G1 D < 10 mm	Adjacent components G2 D < 10 mm	Remote components D > 20 mm	Random input components
N	64	20	44	64	48*
Comparison features of component and motor unit					
RoA (%)	53.2 ± 16.8 (32.1; 100.0)	74.6 ± 12.7 (54.5; 100.0)	43.5 ± 5.8 (32.1; 55.2)	38.5 ± 8.1 (21.1; 65.0)	11.6 ± 2.7 (7.4; 18.8)
D (mm)	5.0 ± 3.1 (0.0; 10.0)	4.8 ± 3.0 (0.0; 9.8)	5.1 ± 3.2 (0.0; 10.0)	23.9 ± 3.0 (20.0; 33.2)	-
Twitch CoV (%)	37.3 ± 14.8 (7.6; 70.6)	22.3 ± 8.5 (7.6; 36.1)	44.2 ± 11.7 (23.1; 70.6)	53.7 ± 22.4 (26.0; 180.7)	80.8 ± 87.5 (38.0; 631.5)
Components' descriptive features					
Firing rate (Hz)	10.7 ± 1.4 (8.4; 15.5)	11.2 ± 1.8 (8.6; 15.5)	10.4 ± 1.2 (8.4; 13.1)	10.0 ± 1.9 (5.6; 15.0)	8.0 ± 12.6 (1.1; 44.9)
IPI CoV (%)	34.1 ± 10.5 (9.4; 58.0)	27.4 ± 10.5 (10.7; 55.8)	37.1 ± 9.1 (9.4; 58.0)	41.2 ± 13.6 (22.9; 91.5)	36.0 ± 51.5 (0.0; 134.1)
TVI variation (m/s)^	9.0 ± 3.4 (3.4; 20.3)	8.9 ± 3.7 (5.0; 20.3)	9.0 ± 3.2 (3.4; 18.1)	9.0 ± 3.4 (3.4; 20.3)	-

*D = distance to needle, RoA = rate of agreement, N = number of components, IPI = inter-pulse-interval, CoV = coefficient of variation, EMG = electromyography, TVI = tissue velocity images. Each cell corresponds to mean ± s.d. (min; max). ^ Values are in terms of e-05 m/s. *16 components were excluded due to no peaks above the 0.35 peak detection criteria (see Methods in main paper).*

Table S2 The number of synchronized EMG-ultrasound measurements per subject and the number of successfully identified MUs in each subject (based on adjacent components G1).

**Subjects**	**# simultaneous EMG-US recordings with MUs identified in EMG**	**# MUs identified in US (based on adjacent components G1)**	**% identified MUs in US (based on adjacent components G1)**
1	7	3	43
2	7	3	43
3	8	5	63
4	6	1	17
5	8	1	13
6	3	0	0
7	8	1	13
8	9	4	44
9	8	2	25
**SUM**	**64**	**20**	**-**

EMG = electromyography, US = ultrasound, MUs = motor units.

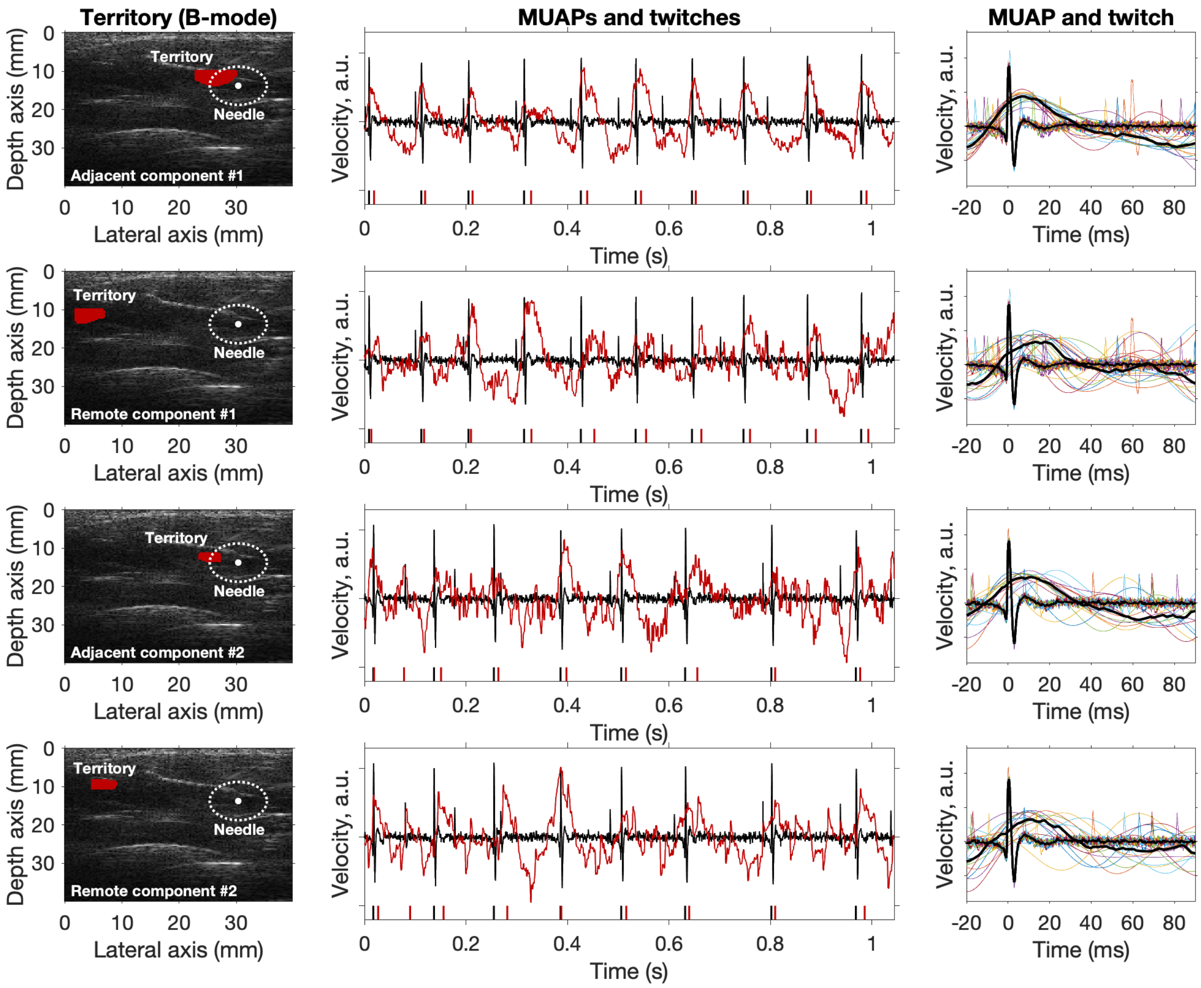

Figure S2 Illustration of one adjacent and one remote component for two different subjects. **First column** Components' territory (red) of biceps brachii cross-section. The concentric needle uptake area are visualized as a dotted circle (white). **Second column** The MUAPs (black), components' twitches (red), and their corresponding firing patterns below (black and red vertical lines). **Third column** The spike-triggered averaged mechanical twitches given the MU firing pattern (from EMG).
